# Molecular identification and biocontrol activity of sugarcane rhizosphere bacteria against red rot pathogen *Colletotrichum falcatum*

**DOI:** 10.1016/j.btre.2019.e00317

**Published:** 2019-02-15

**Authors:** Prittesh Patel, Rushabh Shah, Bhrugesh Joshi, Krishnamurthy Ramar, Amaresan Natarajan

**Affiliations:** C. G. Bhakta Institute of Biotechnology, Uka Tarsadia University, Bardoli, Gujarat, 394 350, India

**Keywords:** Antagonism, Biocontrol, Carbendazim, *Colletotrichum falcatum*, Red rot, Sugarcane

## Abstract

A total of 226 sugarcane rhizosphere-associated bacterial strains from the six different cultivars were screened against three pathogenic strains of *C. falcatum* (cfNAV, cfCHA, and cf8436) for the suppression of red rot disease. On the basis of mycelial growth inhibition in dual culture assay, 26 bacteria were selected for further characterization of morphology, biochemical activity, plant-growth-promoting (PGP) activity, antifungal potential and molecular identity by 16S rRNA gene sequence. On the basis of the 16S rRNA gene sequencing, it was found that the isolates belonged to proteobacteria (13), Firmicutes (10), and Bacteroides (3). The antagonistic bacteria tested for PGP traits revealed that 10 strains were able to solubilize tricalcium phosphate, 11 strains were able to produce siderophore, and 14 strains were able to grow in the N-free medium. The quantitative estimation of indole-3-acetic acid production was ranged from 21.58 to 66.31 μg/mL. On the basis of PGP and biocontrol traits, five strains *Ochrobactrum intermedium* (TRD14), *Acinetobacter* sp. (PK9), *Bacillus* sp. (RSC29 and KR91) and *Escherichia* sp. (VRE34) were further chosen for pot trial under greenhouse conditions on highly susceptible variety *CoC671*. The results showed that the pathogen-inoculated sugarcane plants were able to germinate but died within one month. However, the *CoC671* inoculated with selected biocontrol strains found protected from disease and an increase in plant growth parameters on par with carbendazim fungicides. This study proves that the isolates identified in this study could be used as an alternative to chemical fungicides to control red rot pathogen of sugarcane plants.

## Highlights

• A total of 226 isolates were screened against three strains (cfNAV, cfCHA and cf8436) of *C. falcatum* by dual culture technique. Selected Twenty-Six bacteria characterized of morphology, biochemical activity, PGP activity, antifungal potential and 16S rRNA gene sequence. These isolates belonged to proteobacteria (13), firmicutes (10) and bacterioidetes (03) respectively.
• *Ochrobactrum intermedium* (TRD14), *Acinetobacter* sp (PK9), *Bacillus* sp (RSC29 and KR91) and *Escherichia* sp (VRE34) selected for green house study. The most promising results in suppression of the disease as well as plant growth were observed in treatment withVRE34. The plant height and stem diameter were increased from 13.27 ± 0.67 inches to 24.03 ± 1.40 inches and from 6.07 ± 0.45 mm to 9.87 ± 0.93 mm.
• Isolates identified in this study could be used as an alternative to chemical fungicides to control red rot pathogen of sugarcane plants. However, detailed investigations on their inoculations in the field to confirm its growth promotion potency and biocontrol efficacy under natural environmental and soil conditions shall make these strains as important bioinoculants for integrated disease management of red rot disease in sugarcane.

## 1 Introduction

Sugarcane (*Saccharum officinarum* L.) is one of the economically valuable agricultural crops grown worldwide in tropical and subtropical areas mainly for their sugar source. Among the 110 sugarcane cultivated countries, India and Brazil contribute half of global production [1]. A hemibiotrophic fungal pathogen *Colletotrichum falcatum* causes a major devastating disease in sugarcane [2]. The pathogen initially enters to the plants through the soil and subsequently extends to the stalk by various ways, including borer, which makes the hole in the stem, as well as by other vectors in the field [3]. Infection on leaves may not affect overall yield to a great extent but stalk infection with fungus is very severe as the sugar content is reduced after the infection. During the monsoon period, because of high humidity and lower temperature, the pathogen multiplies rapidly in sugarcane stems. By using stem sucrose for its growth, the pathogen converts sucrose into alcohol and after monsoon period the infected stem starts drying as the alcohol evaporates rapidly. This ultimately reduces the weight of the cane and affects both economies of farmers and the sugar industry. The red rot is a major problem for sugarcane production and is responsible for the abolition of numerous best varieties from the cultivation due to the constant evolution of the newer species [4]. The popular sugarcane variety *CoC671* succumbed to *C. falcatum* during the 1980s in Tamil Nadu, Kerala, Gujarat, Andhra Pradesh, and Pondicherry states of India [5].

The pathogen *C. falcatum* normally resides in the soil as dormant spores and on decayed host plant parts as active saprophytes. The management of the red rot disease in the field is difficult as the genetic make of this fungus changes continuously. There are three main possible ways to control the red rot disease: (1) use of a resistant variety, (2) treatment with fungicides such as carbendazim, and (3) biological control through antagonistic microorganisms. Although the use of resistant varieties is an important approach to control against red rot, the newly released resistant varieties give up to the *C. falcatum* due to the recurrent emergence of its newer variants [6,7]. The use of chemicals to control the phytopathogens results in accumulation of harmful residues in soil, exerting a negative effect on beneficial organisms. In addition, pathogens could acclimatize to surmount and become resistant against these fungicides. Carbendazim is a systemic fungicide generally used to control a range of fungal diseases of agricultural crops [8]. It is moderately stable in water and soil with a half-life of up to 1 year [9]. The persistency of carbendazim in soil and its systemic prevalence in plants can lead to environmental contamination [10]. This causes serious concerns because of its cytotoxicity to mammalian endocrine cells, liver cells, and reproductive tissues [11,12].

There is an increasingly growing demand for biological fertilizers [13]. Control of phytopathogen through biological means involves various mechanisms to inhibit or slow the growth of pathogen [14]. Currently, only a limited number of biocontrol products are available on the market, which makes it desirable to search and study more biocontrol bacteria [15]. The microorganisms that are associated with sugarcane roots may show biocontrol potential against *C. falcatum* pathogens and can play important roles in protecting sugarcane crop. Further, it can be assumed that the crop faces many biotic and abiotic challenges during the long developmental period. The plant-growth-promoting rhizobacteria (PGPR) associated with sugarcane root may be helpful in supporting plant growth by producing various plant-growth-supporting metabolites [16]. PGPR from sugarcane rhizosphere has been reported previously not only to improve plant growth by colonizing rhizosphere but also to suppress *C. falcatum* [3,17]. The development of an effective biological control against *C. falcatum* requires the screening and evaluation of native potential antagonistic bacteria capable of reducing red rot under *in vitro* and *in vivo* conditions. Recently, various bacterial genera including *Pseudomonas*, *Enterobacter*, *Burkholderia*, *Ochrobactrum*, *Gluconacetobacter* and *Bacillus* have been found to be associated with sugarcane rhizosphere with the ability to suppress the *C. falcatum* [3,18,17]. Application of single inoculum, which controls the red rot and simultaneously supports the sugarcane growth, is desirable to reduce the cost of fertilizer and fungicides for farmers. In this regard, efforts have been channelized more toward the growth promotion of plant and simultaneously growth inhibition of pathogens. The aim of this work was to characterize sugarcane root-associated microorganisms for biological control of different strains of *C. falcatum.*

## 2 Materials and methods

### 2.1 Collection of *C. falcatum*

*C. falcatum* strains cfNAV, cfCHA, and cf8436 used in this study were maintained on potato dextrose agar (PDA) slants at 4 °C. Strains cfNAV and cfCHA were chosen according to their higher virulence shown in our previous study [19]. Cf8436 was used as a reference strain obtained from Sugarcane Breeding Institute (Coimbatore, Tamil Nadu, India).

### 2.2 Isolation of sugarcane rhizosphere bacteria

For the isolation of sugarcane rhizosphere bacteria (SRB), rhizosphere soils were collected from 3- to 4-month-old field-grown sugarcane cultivar (*Co86032, Co86249, CoC671, Co814,* and *Co99004*) free from any fungal infection. The composite sample of uprooted rhizosphere soil was transported to the laboratory in an ice box, and isolation of rhizobacteria was carried within 48 h of sample collection. The serial dilution was carried out (10^−1^–10^−6^) with sterile phosphate buffer (pH 6.8) and aliquots of samples were spread on nutrient agar (NA) and Hichrome Bacillus agar, and King’s B agar. All the plates were incubated at 30 ± 2 °C in an incubator for 24 h. Representative bacteria of different morphological types, for example, size, pigmentation, form, and elevation, present on all the plates were selected and purified on NA medium.

### 2.3 Antagonistic activity against *C. falcatum* by SRB

The antagonistic activity of each SRB isolate was studied by dual-culture techniques against the three isolates of *C. falcatum* [20]. Briefly, a small circular plug (5 mm) of each test fungi taken from an actively growing 7-day-old culture on PDA was aseptically placed at 15 mm away from the one end of a sterile 90 mm Petri plate containing PDA. Simultaneously, a loopful of individual overnight grown cultures were separately placed approximately 15 mm away from the opposite end on the same plate. The fungal culture grown on the PDA plate without any bacterial isolate served as control. The experiment was carried out in triplicate. The plates were kept in a plastic bag and incubated for the prescribed period at 30 ± 2 °C in an incubator. At the end of the incubation period, growth was measured. Growth reduction was calculated in relation to the growth of the control, which is as follows:

FGI (%) = ((FGC―FGT)/FGC) × 100

where FGI is the fungal growth inhibition, FGC is the fungal growth in control, and FGT is the fungal growth in treatment.

### 2.4 Effect of carbendazim on *C. falcatum*

The effect of carbendazim on *C. falcatum* was assessed using poisoned food technique [21]. Carbendazim was individually amended to PDA to get fungicide concentrations of 0, 0.01, 0.05, 0.1, and 0.3 with three replications against the three strains of *C. falcatum*. The respective concentrations were achieved by the addition of carbendazim to the pre-autoclaved PDA medium when its temperature was about 40–45 °C. Inoculations were made with an active 5-mm mycelial disk from test isolates in 9-cm Petri plates and incubated at 30 ± 2 °C for seven days. The colony diameter was measured and the growth inhibition was recorded.

### 2.5 Plant-growth-promoting (PGP) traits

To find out the phosphate solubilization potential, Pikovskaya’s agar medium was inoculated at the center of the plate and incubated at 30 ± 2 °C for 96 h [22]. Positive results were observed on the basis of clear halos formed around the bacterial colony. Production of indole-3-acetic acid (IAA) was carried out according to the method given by Bric et al. [23]. SRB cultures grown for 24 h were inoculated into 10 ml sterile 0.1% tryptophan-supplemented LB broth. After incubation at 30 ± 2 °C for 96 h, IAA production was calculated in the culture supernatant using Salkowski reagent. Siderophore production was analyzed using the methodology described by Schwyn and Neilands [24]. The SRB isolates were spot-inoculated on chrome azurol S (CAS) media and incubated for 48 h at 30 ± 2 °C. Positive results were confirmed by the presence of surrounding orange halos due to iron consumption from CAS media. Nitrogen fixation was checked using Jensen media [25].

### 2.6 Gram nature and biochemical characterization

On the basis of biocontrol and PGP traits, selected SRB isolated were subjected to Gram nature and biochemical tests. The biochemical tests such as methyl red, Voges–Proskauer, citrate use, phenyl alanine, nitrate reduction, ammonia production, hydrolysis of casein, lipid, starch and catalase test were performed following the standard protocol [26].

### 2.7 Molecular characterization by 16S rRNA gene and phylogeny

Genomic DNA isolation of selected 26 SRB was carried out by modified cetyl trimethyl ammonium bromide method [27]. After DNA extraction, the integrity and quality of the DNA obtained were checked by 0.8% (w/v) agarose gel electrophoresis and by GelDoc analysis (Bio-Rad). The DNA samples were stored in 100 μl of TE buffer at ―20 °C. 16S rRNA gene amplification was carried out in a thermal cycler using universal bacterial primer set 8F: 5′-AGAGTTTGATCMTGGCTCAG-3′ and 1492R: 5′CGGTTACCTTGTTACGACTT-3′. Thereafter, 50 μl total PCR reaction mixture comprising 200 mM dNTPs, 50 mM each primer, 1 × PCR buffer, 2U Taq polymerase, and 10 ng genomic DNA were prepared. The PCR conditions involved an initial denaturation at 94 °C for 4 min, followed by 35 cycles of 94 °C for 1 min, 52 °C for 1 min, 72 °C for 2 min, and a final extension at 72 °C for 10 min. The isolates were identified for their species level using partial 16S rRNA sequence homology and data were deposited in GenBank (http://www.ncbi.nlm.nih.gov/GenBank/index.html) to obtain accession number. Molecular phylogenetic and evolutionary relationship of all SRB was studied using Mega 7 software. Further, a phylogenetic tree of the *Pseudomonas* and *Bacilli* groups of isolates was separately drawn to compare with the reference strain sequences deposited in GenBank.

### 2.8 Greenhouse pot trial

Pot trial was carried out to select effective biocontrol agents in greenhouse facilities. For this, inoculums of selected five strains, viz., *Ochrobactrum intermedium* (TRD14), *Acinetobacter* sp. (PK9), *Bacillus* sp. (RSC29), *Bacillus* sp. (KR91), and *Escherichia* sp. (VRE34), were prepared by growing bacteria in nutrient broth overnight at 30 ± 2 °C in an incubator shaker. Cultures for seed inoculation were prepared in 0.85% NaCl (saline) after removal of media by centrifugation. The cells were diluted in saline to a final concentration of 1 × 10^6^ cfu/ml. The pathogen was prepared in the form of spore suspension from 10-day-old culture on PDA. Spores were collected in distilled water (DW). Stems having single eye buds were collected from a 7-month-old *CoC671* crop, which is a disease-free highly susceptible sugarcane variety. They were collected from the field and washed properly with DW to remove soil particles on them. The buds were treated with 500 μl bacterial culture and 250 μl pathogen spore suspension at the same time on both open ends of sugarcane stem and then planted in a pot containing autoclaved soil. The effects of the chemical fungicide, carbendazim, were also studied by dipping sugarcane in 0.3 ppm solution for 30 min. before planting. Further, to confirm plant-growth-promoting potential, all five strains were separately inoculated without any pathogen. The inoculated plants were maintained under greenhouse condition at 12:12 h light/dark cycle with regular irrigation. Plants inoculated with DW served as control. The trials were arranged in a randomized complete block design with three replicates. The effects of bacteria were evaluated in terms of plant growth parameter such as height, stem diameter, number of leaves, and condition of the top. Data were recorded on 30 and 60 days after plantation (DAP). The data were examined using SPSS software (SPSS Inc., Chicago, IL) to analyze the variance for a randomized complete block design.

## 3 Results

Although the total numbers of bacterial strains collected using spread plate technique were 226, twenty-six isolates were selected based on biocontrol activity in dual culture assay.

**Fig. 1:**
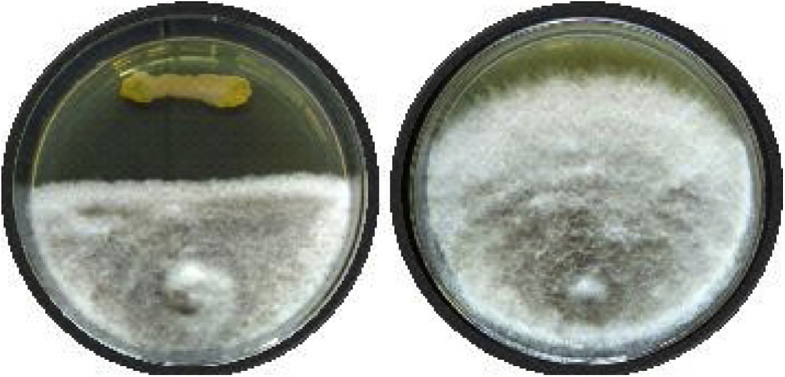
Antifungal activity of *Ochrobactrum intermedium* TRD14 against *C. falcatum* strain cf8436. (Left; Treatment, Right; Control).

The results showed that the antagonistic activity was varied for different strains of *C. falcatum*. The percentage inhibition was ranged from 28.97% to 61.18% for cfNAV, 34.01% to 69.64% for cfCHA, and 28.96% to 53.48% for cf8436 (Table 1 & Fig. 1). In case of *C. falcatum* strain cfNAV, *Ochrobactrum intermedium* TRD14 and *Escherichia* sp. VRE34 has shown maximum inhibition *i.e*. 61.18% and 61.11% respectively. *Escherichia* sp. VRE34 has also shown the highest antagonism against cfCHA (69.64%). Reference strain cf8436 growth was found inhibited maximum when co-inoculated with *Escherichia* sp. VRE34. Out of 26 strains studied 23 strains have sown more than 50% inhibition against one or other *C. falcatum* strain. It can be speculated that higher inhibition recorded in the present study may because of metabolite released from the bacterial strains spread into the PDA continuously. It has been observed that the spread of bacterial growth on media was limited to the inoculation area (Fig. 1). While fungus growth is slow as compare to bacteria so bacterial strains may reach to stationary phase and release as much as metabolites up to seven days. In the case of chemical fungicide carbendazim, the increase in concentration resulted in 100% control (0.3 ppm) of *C. falcatum* mycelia growth. The same concentration was used in pot assay as a positive control.

**Table 1:** Percentage inhibition by biocontrol agents and carbendazim against *C falcatum* isolates. Values in each column with same letter do not differ significantly at P ≤ 0.05 by Duncan’s Multiple Range Test.

Isolate Treatment	%Inhibition against *C falcatum*		
	cfNAV	cfCHA	Cf8436
*Stenotrophomonas acidaminiphila* TRD10	39.52 ^j^	38.26^kl^	34.26^mn^
*Ochrobactrum anthropi* TRD11	31.43 ^k^	34.1^m^	28.96°
*Ochrobactrum intermedium* TRD14	61.18 ^a^	58.8^b^	53.48^b^
*Bacillus safensis* PK1	53.5 ^b^	40.47^ijk^	43.31^ghi^
*Bacillus megaterium* PK2	52.29^bc^	42.67^ghi^	46.05^fg^
*Sphingobacterium thalpophilum* PK6	41.37 ^ij^	37.65^klm^	36.71^lm^
*Acinetobacter* sp. PK9	60.44 ^a^	51.63 ^de^	49.75^de^
*Acinetobacter* sp. PK10	50.46 ^cd^	53.32^cd^	49.61 ^de^
*Stenotrophomonas acidaminiphila* RSC6	51.04 ^cd^	41^hij^	47.68^ef^
*Sphingobacterium thalpophilum* RSC24	48.64 ^de^	42.81^gh^	43.82^gh^
*Escherichia* sp. RSC25	28.97 ^k^	40.06^ijk^	37.54^kl^
*Bacillus* sp. RSC29	51.07 ^bcd^	57.05^b^	50.09^cd^
*Enterobacter* sp. RSC32	44.37 ^gh^	44.27^gh^	40.43^ijk^
*Bacillus* sp. KR91	58.65 ^a^	56.89^bc^	53.12^bc^
*Cronobacter muytjensii* VRE6	42.58 ^ghi^	45.05^g^	36.22^lm^
*Enterobacter cloacae* VRE7	52.38 ^bc^	39.16^jkl^	38.67^jkl^
*Pseudomonas* sp. VRE8	41.27 ^ij^	45.03^g^	44.42^fg^
*Bacillus thuringiensis* VRE11	45.34 ^fg^	46.02 ^fg^	36.25^lm^
*Pseudomonas aeruginosa* VRE12	54.17 ^b^	49.95 ^de^	45.38^fg^
*Sphingobacterium* sp. VRE29	48.55 ^de^	36.11 ^lm^	34.78^mn^
*Escherichia* sp. VRE34	61.11^a^	69.64 ^a^	58.96^a^
*Pseudomonas* sp. S1	41.68 ^hij^	37.7^klm^	32.51^n^
*Pseudomonas plecoglossicida* S2	47.27 ^ef^	51.93 ^de^	39.13^jk^
*Pseudomonas* sp. S4	45.24 ^fg^	38.63^kl^	39.1^jkl^
*Bacillus safensis* B1	44.37^gh^	45.12^g^	41.06^hij^
*Bacillus subtilis* C1	50.46 ^cd^	48.85 ^ef^	51.2^bc^
*Carbendazim (0.01 ppm)	20.22	22.37	27.98
*Carbendazim (0.05 ppm)	59.39	50.84	61.51
*Carbendazim (0.1 ppm)	80.26	79.15	83.06
*Carbendazim (0.3 ppm)	100	100	100

* Fungicide.

All twenty-six isolates were characterized for their morphology and metabolic activities (Table 2). The Gram’s reaction showed that out of 26 isolates, 7 were positive and the rest was negative. The biochemical tests of antagonistic bacteria showed that none of the isolates were positive for phenyl alanine and Voges–Proskauer test. The results of other experiments varied for each isolate. However, 13 bacteria were found to be capable of reducing nitrate and 6 producing ammonia.

**Table 2:** Biochemical characterization of sugarcane rhizospheric microbes. MR- Methyl Red, VP- Voges Proskauer, PA- Phenyl Alanine, NH_3_-Ammonia.

SRB	Gram reaction	MR	VP	Citrate	PA	Urea	Nitrate	NH_3_	Amylase	Protease	Lipase	Catalase
TRD10	–	–	–	–	–	–	+	+	–	–	–	–
TRD11	–	–	–	–	–	+	–	–	–	+	–	–
TRD14	–	–	–	–	–	+	–	+	–	–	–	+
PK1	+	–	–	+	–	–	–	–	–	+	+	–
PK2	+	–	–	–	–	–	+	–	+	–	–	–
PK6	–	+	–	–	–	–	+	–	–	–	–	–
PK9	–	–	–	–	–	–	–	+	+	+	–	–
PK10	–	–	–	–	–	–	+	–	–	–	–	+
RSC6	–	–	–	+	–	+	–	–	–	+	–	–
RSC24	–	–	–	–	–	–	+	–	–	–	+	–
RSC25	–	–	–	+	–	–	–	–	+	–	–	–
RSC29	+	–	–	–	–	–	–	+	–	–	–	+
RSC32	–	–	–	–	–	–	+	–	–	–	+	–
KR91	+	+	–	–	–	–	+	–	–	+	–	+
VRE6	–	–	–	+	–	–	–	+	–	–	–	–
VRE7	–	–	–	–	–	–	+	–	–	–	–	–
VRE8	–	–	–	–	–	+	–	–	–	+	+	–
VRE11	+	–	–	–	–	–	–	–	+	+	–	–
VRE12	–	–	–	–	–	–	+	–	–	+	–	–
VRE29	–	+	–	–	–	–	+	–	–	–	–	–
VRE34	–	–	–	–	–	–	–	+	–	+	–	+
S1	–	–	–	–	–	+	–	–	–	+	–	–
S2	–	–	–	+	–	–	–	–	+	–	–	–
S4	–	+	–	–	–	–	+	–	–	–	+	–
B1	+	–	–	–	–	+	–	–	+	–	–	+
C1	+	–	–	+	–	–	+	–	–	+	–	–

–= Negative += Positive.

The identification of antagonistic bacteria based on 16S rRNA gene sequencing showed the presence of 10 different genera. These isolates belonged to proteobacteria (13), Firmicutes (10), and Bacteroides (3). It is interesting to note that a diverse group of bacteria could be isolated from the rhizosphere soils of sugarcane (Table 3).

**Table 3:** List of SRB isolated from sugarcane rhizosphere.

Sr NO	Isolate code	Host Cultivar	Place of collection	% Homology	Identified as	Accession Number
1	TRD10	Co 86032	Mahuva	99	*Stenotrophhomonas acidaminiphilia*	MF351813
2	TRD11	Co 86032	Mahuva	99	*Ochrobactrum anthropi*	KY672866
3	TRD14	Co 86032	Mahuva	99	*Ochrobactrum intermedium*	MF351814
4	PK1	Co 86249	Timbarva	99	*Bacillus safensis*	KU867835
5	PK2	Co 86249	Timbarva	99	*Bacillus megaterium*	KU867836
6	PK6	Co 86249	Timbarva	99	*Sphingobacterium thalpophilum*	KU867842
7	PK9	Co 86249	Karachka	99	*Acinetobacter* sp.	KX168053
8	PK10	Co 86249	Karachka	99	*Acinetobacter* sp.	KX168037
9	RSC6	Co 671	Navsari	99	*Stenotrophomonas acidaminiphila*	KU867837
10	RSC24	Co 671	Navsari	99	*Sphingobacterium thalpophilum*	KU867848
11	RSC25	Co 671	Madhi	99	*Escherichia* sp.	KX228402
12	RSC29	Co 671	Madhi	99	*Bacillus* sp.	KX181401
13	RSC32	Co 671	Madhi	99	*Enterobacter* sp.	KX168052
14	KR91	Co 8145	Rayam	99	*Bacillus* sp.	KX168055
15	VRE6	Co 94004	Vyara	98	*Cronobacter muytjensii*	KU867847
16	VRE7	Co 94004	Vyara	99	*Enterobacter cloacae*	KU867838
17	VRE8	Co 94004	Varad	99	*Pseudomonas* sp.	KX168038
18	VRE11	Co 94004	Vyara	99	*Bacillus thuringiensis*	KU867844
19	VRE12	Co 94004	Vyara	99	*Pseudomonas aeruginosa*	KU867839
20	VRE29	Co 94004	Vyara	99	*Sphingobacterium* sp.	KU867840
21	VRE34	Co 94004	Varad	99	*Escherichia* sp.	KX228403
22	S1	Co 86002	Rajpara	99	*Pseudomonas* sp.	KX168054
23	S2	Co 86002	Timbarva	99	*Pseudomonas plecoglossicida*	KU867841
24	S4	Co 86002	Timbarva	99	*Pseudomonas* sp.	KU867843
25	B1	Co 86002	Timbarva	99	*Bacillus safensis*	KU867845
26	C1	Co 86002	Timbarva	99	*Bacillus subtilis*	KU867846

**Fig. 2:**
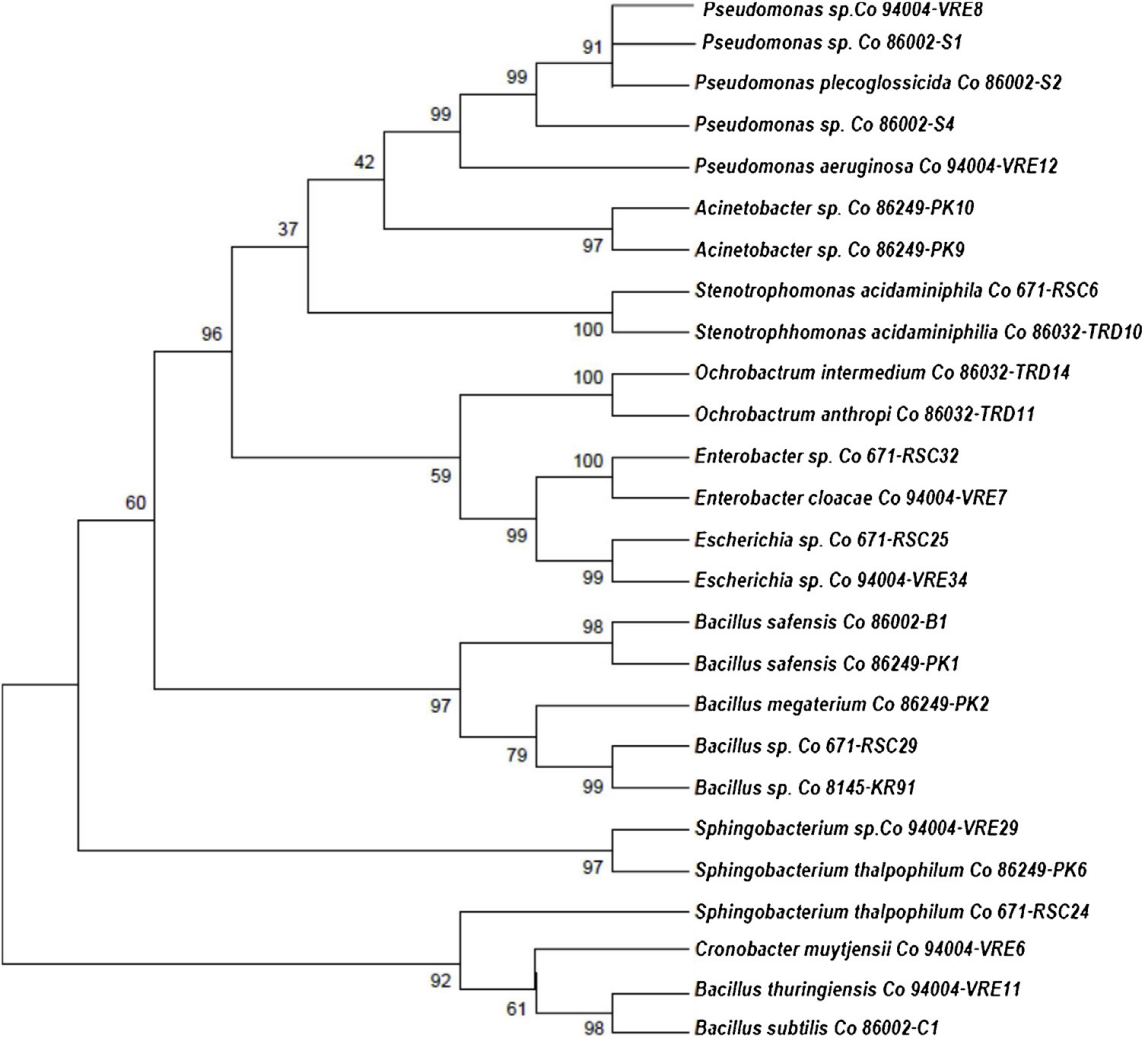
A phylogram derived from sequences of 16S rDNA region of sugarcane rhizospheric isolates collected in the present study. Numbers on nodes represent bootstrap values (%) from 500 replicates. A phylogenetic tree was constructed using MEGA 7.0.

The evolutionary history and molecular diversity were inferred using the UPGMA method. The optimal tree with the sum of branch length = 4.61531710 is shown. The percentage of replicate trees in which the associated taxa clustered together in the bootstrap test (500 replicates) is shown next to the branches. The evolutionary distances were computed using the Kimura 2-parameter method and are in the units of the number of transitional substitutions per site. The rate variation among sites was modeled with a gamma distribution (shape parameter = 1). All ambiguous positions were removed for each sequence pair. There were a total of 1544 positions in the final dataset. The results of the phylogenetic analysis showed one major group with five *Pseudomonas* strains (*Pseudomonas* sp. (VRE8), *Pseudomonas* sp. (S1), *P. plecoglossicida* (S2), *Pseudomonas* sp. (S4), and *P. aeruginosa* (VRE12)) including three from Co 86002 grouped as cluster 1. Cluster 2 presented two strains (PK9 andPK10) from *Co86249*. Two strain of *Stenotrophomonas acidaminiphila* (RSC6 and TRD10) were grouped under cluster 3. TRD14 and TRD11 were grouped in cluster 4. *Enterobacter* sp. and *Escherichia* sp. were grouped under cluster 5. Clusters 6 and 8 presented isolates belonging to genus *Bacillus*. Cluster 7 consisted of four isolates belonging to the order *Sphingobacter* (Fig. 2).

The antagonistic bacteria tested for PGP traits showed that 10 isolates were able to solubilize tricalcium phosphate in the medium. Eleven strains were able to produce siderophore and 14 strains were able to grow in the N-free medium. The quantitative estimation showed that all strains were able to produce IAA in certain quantities, which ranged from 21.58 to 66.31 μg/ml (Table 4).

**Table 4:** Plant growth promoting characters of sugarcane rhizospheric microbes.

SRB	IAA Production (μg/ml)	P Solubilization	N2 Fixation	Siderophore
*Stenotrophomonas acidaminiphila* TRD10	21.58	–	–	+
*Ochrobactrum anthropi* TRD11	34.28	–	–	–
*Ochrobactrum intermedium* TRD14	66.31	+	+	–
*Bacillus safensis* PK1	39.68	–	–	–
*Bacillus megaterium* PK2	44.28	–	+	+
*Sphingobacterium thalpophilum* PK6	43.73	–	+	+
*Acinetobacter* sp. PK9	63.79	+	–	+
*Acinetobacter* sp. PK10	59.09	+	+	–
*Stenotrophomonas acidaminiphila* RSC6	56.98	+	+	–
*Sphingobacterium thalpophilum* RSC24	23.74	+	+	–
*Escherichia* sp. RSC25	54.28	+	+	–
*Bacillus* sp. RSC29	65.66	+	+	–
*Enterobacter* sp. RSC32	56.98	+	+	–
*Bacillus* sp. KR91	60.36	–	+	+
*Cronobacter muytjensii* VRE6	36.31	–	+	–
*Enterobacter cloacae* VRE7	34.54	–	–	–
*Pseudomonas* sp. VRE8	43.86	+	–	–
*Bacillus thuringiensis* VRE11	27.11	–	–	–
*Pseudomonas aeruginosa* VRE12	54.95	–	–	–
*Sphingobacterium* sp. VRE29	25.90	–	–	–
*Escherichia* sp. VRE34	51.16	+	+	+
*Pseudomonas* sp. S1	53.94	–	–	+
*Pseudomonas plecoglossicida* S2	42.38	–	–	+
*Pseudomonas* sp. S4	47.11	–	+	+
*Bacillus safensis* B1	33.40	–	–	+
*Bacillus subtilis* C1	37.40	–	+	+

–= Negative + = Positive.

On the basis of antagonistic and PGP properties, five strains were tested *in vivo* against three strains of *C. falcatum*. The results revealed that TRD14was most effective in controlling the pathogenicity of cfNAV and promoted sugarcane height up to 18.33 ± 1.08 in. on 60 DAP compared to control (15.03 ± 2.61 in.) (Table 5). Furthermore, sugarcane treated with TRD14 also experienced an increase in stem diameter from 6.37 ± 0.50 mm (30 DAP) to 10.27 ± 0.45 mm (60 DAP), and the condition of the top was green even after two months. In the case of PK9 and RSC29, when challenged with *C. falcatum* strains, it was found that plant height and stem diameter were not significantly supported and sugarcane was started drying after 45 days, but in absence of any red rot pathogen both the strains helped increase the stem height up to 16.63 ± 1.2 and 16.63 ± 2.3 in., and stem diameter up to 7.27 ± 0.45 and 8.83 ± 0.70 mm. Similarly, KR91 without pathogen challenge supported maximum plant height and stem diameter up to 21.53 ± 1.60 in. and 9.07 ± 0.76 mm 60DAP and the condition of the top was green. Further, inoculation of KR91 showed a good biocontrol activity against cf8436 in which the plant height and stem diameter were more than doubled, that is, from 9.63 ± 0.85 in. (30 DAP) to 21.10 ± 1.7 in. (60 DAP). The most promising results in suppression of the disease as well as plant growth were observed in treatment withVRE34. The plant height and stem diameter were increased from 13.27 ± 0.67 in. to 24.03 ± 1.40 in. and from 6.07 ± 0.45 mm to 9.87 ± 0.93 mm. VRE34 supported good plant growth in treatment with all pathogens. In the case of carbendazim treatment, plants showed good growth but leaves almost became pigment-less on the 60th day against cfCHA and cf8436. It was observed that the sugarcane growth was almost stopped on 30 DAP when inoculated with red rot pathogens. In addition, stem diameter was found to decrease from 30 to 60 DAP because of plant death followed by dryness of tissues. This directly reflects the severity of red rot pathogen. There was no significant difference in the number of leaves, and in the majority of cases, it was 3–4 on 60 DAP, especially in pathogen-inoculated pots where plants died within a month and the number of leaves produced was only 2.

**Table 5:** Evaluation of the five SRB for their antagonistic and growth promotion potential against *C. falcatum* in sugarcane CoC 671 under green house conditions. Values in each column with same letter do not differ significantly at P ≤ 0.05 by Duncan’s Multiple Range Test.

Treatment	Plant Height (inches)		Stem Diameter (mm)		No of Leaves		Condition of plant (60 DAP)
	30 DAP	60 DAP	30 DAP	60 DAP	30 DAP	60 DAP	
Control	10.17 ^d^	15.03 ^gh^	4.77 ^de^	7.17^fg^	3	4	G
TRD14	13.17 ^b^	23.10 ^ab^	6.37 ^ab^	10.27 ^a^	3	4	G
TRD14 X cfNAV	5.17 °	18.33 ^de^	4.13^gh^	8.10 ^cd^	4	4	G
TRD14 X cfCHA	9.67 ^f^	10.43 ^l^	5.07 ^cd^	6.37^gh^	2	3	G
TRD14 X cf8436	3.37 ^p^	6.23^n^	3.87 ^hi^	6.10^ij^	3	4	G
PK9	10.8 ^cd^	16.63 ^ef^	5.77 ^ab^	7.27^fg^	3	5	G
PK9 X cfNAV	10.87 ^cd^	10.77 ^k^	4.93 ^de^	5.63 ^jk^	3	3	G
PK9 X cfCHA	8.93 ^i^	11.13 ^jk^	4.23^gh^	4.53 ^l^	3	3	G
PK9 X cf8436	8.37 ^j^	11.60 ^jk^	5.70 ^ab^	5.93 ^k^	3	3	G
RSC29	13.77 ^b^	16.63 ^ef^	5.37 ^cd^	8.83^bc^	4	5	G
RSC29 X cfNAV	8.27 ^jk^	9.40^lm^	4.80^de^	6.57^gh^	3	3	G
RSC29 X cfCHA	8.83 ^i^	10.47^l^	3.93 ^hi^	5.17 ^kl^	3	3	G
RSC29 X cf8436	10.70 ^cd^	12.90^ij^	4.20^gh^	7.23^fg^	3	3	G
VRE34	13.27 ^b^	24.03 ^a^	6.07 ^ab^	9.87 ^a^	4	5	G
VRE34 X cfNAV	8.90 ^i^	13.30 ^hi^	4.27^gh^	7.83 ^ef^	2	4	G
VRE34 X cfCHA	11.57 ^c^	14.27 ^hi^	3.67 ^i^	6.17 ^ij^	3	3	G
VRE34 X cf8436	7.43 ^l^	19.33 ^cd^	3.23 ^k^	6.53^gh^	3	4	G
KR91	17.37 ^a^	21.53 ^bc^	6.63 ^a^	9.07 ^b^	4	5	G
KR91 X cfNAV	6.43^n^	8.07^mn^	5.77^ab^	5.97 ^jk^	2	3	G
KR91 X cfCHA	7.83^kl^	13.37 ^hi^	5.33 ^cd^	5.73^kl^	2	3	G
KR91 X cf8436	9.63^g^	21.10 ^bc^	5.27 ^cd^	9.03 ^b^	2	4	G
Carbendazim	8.37^j^	15.53^fg^	5.47^bc^	8.70^bc^	3	4	G
Carbendazim X cfNAV	9.40 ^h^	14.40 ^gh^	4.67^ef^	7.93 ^de^	3	4	G
Carbendazim X cfCHA	7.0^m^	9.43 ^lm^	5.60^bc^	6.37 ^i^	3	3	G
Carbendazim X cf8436	11.23 ^cd^	14.77 ^gh^	5.77^ab^	7.03 ^g^	3	3	G
cfNAV	7.2 ^lm^	7.43^mn^	4.47 ^fg^	4.37 ^l^	2	2	D
cfCHA	3.73 ^p^	3.97 °	3.47 ^i^	3.40 ^m^	2	2	D
Cf8436	4.0 ^op^	4.07 °	3.33 ^jk^	3.23 ^m^	2	2	D

^*^G- Green, D-Dry.

## 4 Discussion

The present study was successful in selecting 26 most promising bacteria from sugarcane rhizosphere that can be a useful component of integrated disease management. Microorganisms that can colonize the rhizosphere and show biocontrol potential may have an important role for crop protection against soil-borne plant pathogens [28]. Although rhizobacteria-mediated biocontrol is an easy and eco-friendly way but best results can be obtained only with its host-specific selection. In the case where the pathogen is diverse in nature, for example *C. falcatum*, it becomes necessary to assess a large number of biocontrol agents. The *C. falcatum* variants are referred as pathotypes and found to show high compatibility with the host variety. Further, there would be possibility of a variety of microorganisms associated with roots of different cultivars. In view of this, in the present study different sugarcane varieties were selected in the range of resistance to highly susceptible categories for the isolation of rhizosphere bacteria which have the ability to control highly virulent red rot strains cfCHA, cfNAV, and cf8436 [19].

According to Essghaier et al. [29] PGP bacteria can enhance plant growth through a broad range of activities such as IAA production, phosphate solubilization, siderophore production, and nitrogen fixation. Although many studies on the PGP activities of the sugarcane rhizobacteria have been reported till date, only a few of them have reported both PGP and biocontrol. In recent years, different rhizobacteria including members of the genus *Acinetobacter* and *Klebsiella* have been reported from the sugarcane to possess PGP properties [16,[30], [31], [32]].

In this study, we have reported the toxicity of carbendazim against *C. falcatum* under *in vitro* conditions by poison food technique. It was found that only 0.3 ppm carbendazim is sufficient to completely seize spore germination and mycelial growth. López-Herrera and Zea-Bonilla [33] found benomyl, carbendazim, and thiophanate methyl at 0.5 μg/mL totally inhibited mycelial growth of *Rosellini anecatrix*. Similar results were noted by Waraitch [34], who reported that carbendazim was most effective against *C. falcatum*. Although chemical control of *C. falcatum* is intensive and effective, it poses problems to the human health as well as environment. Biocontrol with PGP activity by native bacterial strain would be best inoculums for high yield of sugarcane with minimum cost. Among all the five strains studied, TRD14 was found highly effective with IAA production, phosphate solubilization, nitrogen fixation, more than 50% inhibition again all three tested pathogens, and increased growth parameters in pot assay. So, TRD14 holds immense potential for future use in the management of red rot disease. Muangthong et al. [35] isolated *O. intermedium* from industrial sugarcane (UT3R1) varieties and reported them to have N fixation capacity. Previously, Bajoria et al. [36] reported the antifungal activity of *O. intermedium* against *Macrophomina phaseolina* and *Fusarium oxysporum*. Members of the genus *Bacillus* are large painstaking microbial factories for the release of a monstrous array of biologically active metabolites possibly controlling the growth of phytopathogen. In this study, we have characterized seven *Bacilli* strains with antagonistic potential in detail, and among them, two strains viz., RSC29 and KR91, were evaluated for biological control under greenhouse condition. *B. safensis* (PK1) and *B. megaterium* (PK2) have shown more than 50% mycelial growth inhibition for cfNAV. Our results are in accordance with Hassan et al. [37] who reported two antagonistic strains *B. subtilis* NH-100 and *Bacillus* sp. NH-217 against *C. falcatum.* Antagonistic strains of the genus *Bacillus* are advantageous over other biocontrol agents in various ways, as they are omnipresent in soils, have excessive sporulation, have prolonged shelf life, and enhance plant nutrition. Their efficiency in controlling many plant diseases has repeatedly been shown by many researchers [38,39]. Further, results obtained with VRE34 also indicated the possible application as biofertilizer and biopesticide. The sugarcane treated with only pathogens showed severe disease condition and plants died within a month. Therefore, the results showed that these five bacterial isolates might have the potential to be developed as a promising commercial biological control agent in the future. Biocontrol of *C. falcatum* by effective antagonistic of microbes in a controlled laboratory or greenhouse conditions has been reported in many studies [3,40]. However, together with other tests need to be done, such as field experiments and assessing suitability in a fermenter for large-scale production for commercial biofertilizer, survival potential, root colonization, adequate dose, chemical compatibility, the symbiotic effect on other community and economic viability.

In conclusion, irrespective of the mechanisms underlying interactions between sugarcane plant and endophytic pathogen, native rhizobacteria studied were capable to control *C. falcatum* under both *in vitro* and *in vivo* conditions. However, detailed investigations on their inoculations in the field to confirm its growth promotion potency and biocontrol efficacy under natural environmental and soil conditions shall make these strains as important bioinoculants for integrated disease management of red rot disease in sugarcane.

## Conflict of interest statement

The authors report no conflicts of interest. The authors alone are responsible for the content and writing of the paper.
